# TMAO and Gut Microbial-Derived Metabolites TML and γBB Are Not Associated with Thrombotic Risk in Patients with Venous Thromboembolism

**DOI:** 10.3390/jcm11051425

**Published:** 2022-03-04

**Authors:** Marina Canyelles, Melania Plaza, Noemí Rotllan, Dolors Llobet, Josep Julve, Sergi Mojal, Maribel Diaz-Ricart, José Manuel Soria, Joan Carles Escolà-Gil, Mireia Tondo, Francisco Blanco-Vaca, Joan Carles Souto

**Affiliations:** 1 Institut de Recerca de l’Hospital Santa Creu i Sant Pau, Institut d’Investigacions Biomèdiques IIB Sant Pau, 08041 Barcelona, Spain; mcanyelles@santpau.cat (M.C.); mplazas@santpau.cat (M.P.); nrotllanv@santpau.cat (N.R.); mllobetl@santpau.cat (D.L.); jjulve@santpau.cat (J.J.); smojal@santpau.cat (S.M.); jsoria@santpau.cat (J.M.S.); jescola@santpau.cat (J.C.E.-G.); jsouto@santpau.cat (J.C.S.); 2 Department of Clinical Biochemistry, Hospital de la Santa Creu i Sant Pau, IIB Sant Pau, 08041 Barcelona, Spain; 3 CIBER de Diabetes y Enfermedades Metabólicas Asociadas (CIBERDEM), 28029 Madrid, Spain; 4 Department de Bioquímica i Biologia Molecular, Universitat Autònoma de Barcelona, 08041 Barcelona, Spain; 5 Unit of Thrombosis and Hemostasis, Hospital de la Santa Creu i Sant Pau, 08041 Barcelona, Spain; 6 Hematopathology, Department of Pathology, Centre de Diagnostic Biomedic (CDB), Hospital Clinic de Barcelona, Institut d’Investigacions Biomediques August Pi i Sunyer (IDIBAPS), Universitat de Barcelona, 08007 Barcelona, Spain; mdiaz@clinic.cat; 7 Genomics of Complex Diseases Group, Research Institute of Hospital de la Santa Creu i Sant Pau, IIB Sant Pau, 08041 Barcelona, Spain

**Keywords:** trimethylamine N-oxide, TMAO, γBB, TML, liquid chromatography–mass spectrometry, venous thromboembolism

## Abstract

Background: The present work evaluates the association between circulating concentrations of Trimethylamine-N-oxide (TMAO), gamma butyrobetaine (γBB), and trimetyllisine (TML) in controls and patients with venous thromboembolism (VTE) with coagulation parameters. Methods: The study involved 54 VTE patients and 57 controls. Platelet function, platelet hyperreactivity, platelet adhesiveness, thrombosis-associated parameters, and thrombin generation parameters were studied. Plasma TMAO, γBB, and TML determination was performed using an ultra-high-performance liquid chromatography system coupled with mass spectrometry. Results: No differences were found for TMAO, γBB, or TML concentrations between controls and VTE patients. In thrombin generation tests, TMAO, γBB, and TML showed a positive correlation with lag time and time to peak. TMAO, γBB, and TML negatively correlated with peak height. No significant differences were observed regarding TMAO, γBB, and TML concentrations between the two blood withdrawals, nor when the control and VTE patients were analyzed separately. No correlation was observed between these gut metabolites and platelet function parameters. Conclusions: No differences were found regarding TMAO, γBB, and TML concentrations between the control and VTE groups. Some correlations were found; however, they were mild or went in the opposite direction of what would be expected if TMAO and its derivatives were related to VTE risk.

## 1. Introduction

Trimethylamine-N-oxide (TMAO) is a small organic compound formed in the liver and produced by the action of hepatic flavin monooxygenase 3 (FMO3) on trimethylamine (TMA). In turn, TMA is generated by the action of gut microbiota using precursors from the diet as choline or other choline-containing compounds, betaine, or L-carnitine, as a part of microbial-mammalian metabolism [1]. TMAO can accumulate as an osmolyte compound in the tissues or be cleared by the kidneys through urine.

TMAO garnered attention some years ago due to its association with cardiovascular diseases (CVD), specifically atherothrombotic events in the context of myocardial infarction and stroke [2,3,4]. Accordingly, a door for the identification of novel microbial and mammalian metabolic pathways was opened. Other than TMAO, γ-butyrobetaine (γBB), produced from dietary L-carnitine, and its precursor trimethyllysine (TML) have also been linked with cardiovascular mortality in patients with carotid atherosclerosis [5].

Bacteria can participate in thrombosis etiology through various mechanisms, including TMAO production. Studies using animal models have provided evidence that TMAO promotes platelet responsiveness to multiple agonists by stimulating Ca2+ release from intracellular stores, heightening thrombosis potential [6]. A recent in vitro study of human coronary endothelial cells showed that TMAO promotes thrombosis by increasing tissue factor expression and activity [7]. In this regard, plasma TMAO levels have been associated with a higher risk of thrombotic events in human subjects [8]. Altogether, these effects translate into an increased thrombogenicity in a microbiota-dependent manner.

Venous thromboembolism (VTE), which includes deep vein thrombosis (DVT) and pulmonary embolism (PE), is the third cause of cardiovascular death worldwide [9]. Despite current evidence, the role of TMAO exposure in human thrombosis remains unclear. In this regard, a prospective cohort study of 859 patients with acute VTE found that TMAO was not a clear predictor, as higher levels of TMAO positively correlated with death but not with VTE recurrence [10]. Overall, the role of gut microbiome-derived metabolites in the context of clinical VTE deserves further attention.

Our hypothesis is that higher TMAO concentrations are associated with an increased risk of VTE. Therefore, our aims were, first, to evaluate the circulating concentrations of TMAO and two of its precursors, γBB and TML, in controls and patients with prior VTE events and, second, to study the correlation of TMAO, γBB, and TML with coagulation parameters.

## 2. Materials and Methods

### 2.1. Study Population

A final number of 111 individuals were enrolled in the study, including 66 females (59.5%) and 45 males (40.5%) with a mean age of 61.7 years (16.7). This is a sub-cohort of the original RETROVE (Riesgo de Enfermedad TROmboembólica VEnosa) cohort. Briefly, the RETROVE study was a case–control study that included 400 consecutive patients with VTE (older than 18 years) and 400 healthy volunteers without history of VTE who served as controls. The objective of the RETROVE Study was to identify biomarkers for VTE and to establish mathematical algorithms to predict its risk [11]. The subgroup included in our study had 54 patients with VTE, randomly selected from the original cohort, and 57 controls, matched with the VTE cohort for age and gender. Blood samples from the VTE patients were taken at least 6 months after the thrombosis episode in order to minimize the influence of the acute phase. None of the participants was using oral anticoagulants, heparin, or antiplatelet therapy at the time of the blood withdrawal. The period of patient recruitment was from 2012 to 2016. A first blood sample was obtained at the time of recruitment (baseline), and a second independent blood sample (endpoint) was obtained after a median follow-up of 5 years (3–7) (min–max), representing a total of 222 samples studied.

The diagnosis of VTE was based on Doppler ultrasonography, tomography, magnetic resonance, arteriography, phlebography, and pulmonary gammagraphy. Inclusion criteria included all types of thrombosis except those related to cancer. VTE events were classified as spontaneous or non-spontaneous (one or more provoking factors within three months prior to an event), as shown in Table 1. Provoking factors included surgery, immobilization, pregnancy or puerperium, oral contraceptives, prothrombotic non-neoplastic diseases, and other circumstances [12].

The study was approved by the Sant Pau Ethics Committee, nr. 04/2012, 7 February 2012, following the standards for medical research in humans recommended by the Declaration of Helsinki. All participants gave written informed consent before enrollment in accordance with the guidelines of the local ethics committee.

### 2.2. Coagulation Parameters

#### 2.2.1. Platelet Function Analysis (PFA)

For the PFA, blood samples were collected in the antecubital vein and anticoagulated with 1/10 volume of 0.129 mol/l sodium citrate (BD Vacutainer Becton, Dickinson, and Company, New Jersey, USA). Platelet-rich plasma (PRP) was obtained by centrifugation at 160× *g* for 10 min. The analysis was performed using a PFA-100 analyzer. The 54 patients were selected from those with lower percentiles (<10%) from platelet occlusion times measured by PFA-100. The citrated whole blood samples were transferred to the reservoir of the disposable test cartridges (PFA_ADP and PFA_EPI) inserted into the instrument, and both closure times (CT) were recorded.

#### 2.2.2. Platelet Hyperreactivity (PHR) Analysis

PHR is a phenotype related to sticky platelet syndrome and characterized by an increase in dose-dependent platelet aggregation patterns activated by agonists adenosine 5′-diphosphate (ADP) and epinephrine (EPI) at low concentrations in PRP. The test was performed by light transmission aggregometry (LTA) (Biometa, Helena), measuring the percentage of maximal aggregation and the percentage area under the curve (AUC). The agonists were diluted with physiological saline. ADP and EPI at 0.5 mmol/L were used to differentiate between hyper-responders and hypo-responders. Higher concentrations of ADP and EPI (2 mmol/l and 10 mmol/l, respectively) were used as positive controls.

#### 2.2.3. Platelet Adhesiveness (PA)

PA was evaluated using two experimental approaches. The first was the IMPACT system, which uses whole blood samples on a plastic surface (750 rpm) and microfluidic devices to explore platelet adhesion on collagen type I (shear rate: 800 s^−1^). The second approach used was 2D and 3D evaluation by confocal microscopy. The studied parameters were aggregate size (AS), surface covered (SC), and number of objects (OB).

#### 2.2.4. Thrombosis-Associated Parameters

Thrombosis-associated parameters included A disintegrin-like and metalloprotease with thrombospondin type 1 motif no. 13 (ADAMTS13), ristocetin cofactor (RIS), and von Willebrand factor (VWF). The ADAMTS13 antigen was determined using a TECHNOZYM^®^ADAMTS13 ELISA kit (Technoclone GmbH, Vienna, Austria) according to the manufacturer’s instructions. The VWF antigen was determined by the commercial VWF Antigen test REAADS kit (Broomfield, CO, USA). A RIS assay with platelets reproduces in vitro the ability of VWF to interact with the platelet receptor glycoprotein GPIb-IX-V complex, in the presence of ristocetin (Von Willebrand Factor Ristocetin Cofactor Activity, Bedford, MA, USA).

#### 2.2.5. Thrombin Generation Parameters

Thrombin generation was measured in platelet-poor plasma (PPP), which was obtained by centrifugation at 2000× *g* for 20 min. Thrombin generation measurement was performed using a semi-automated calibrated automated thrombogram (CAT; Thrombinoscope, Diagnostica Stago, Asnières, France). The measured parameters were lag time (LAG), time to peak (TTP), peak height (PEAK), and endogen thrombin potential (ETP).

### 2.3. Plasma TMAO, γBB, and TML Determination

To determine TMAO and its metabolite (γBB and TML) concentrations, blood samples were collected in EDTA tubes with subsequent centrifugation (10 min at 10,000× *g*); plasma aliquots were stored at –80 °C until analysis. TMAO and its derivatives were determined using an ultra-high performance liquid chromatography system coupled with mass spectrometry (uhLC-MS). The internal standards (IS) used for the study were d3-methylcarnitine (d3-MeCar) to quantify the γBB and TMAO concentrations and ^13^C3-TML for TML, both at 5 ppm. A human serum pool spiked with standards was used to prepare the calibration curves (0–250 µM for TMAO, 0–25 µM for γBB, and 0–20 µM for TML). 

Briefly, 25 µL of human plasma and 300 µL of acetonitrile:methanol:water (5:4:1; *v*:*v*:*v*), containing the two IS, were mixed and vortexed for 20 s. After 30 min of re-equilibration on ice, the samples were centrifuged at 25,100× *g* for 10 min at 4 °C. The supernatant was transferred to a specific vial prior to LC-MS analysis. The extracts were analyzed using an uhLC system coupled with a 6490 triple-quadrupole mass spec-trometer (QqQ, Agilent Technologies, CA, USA) with an electrospray ion source (LC-ESI-QqQ) working in positive mode. An Acquity UPLC BEH HILIC column (1.7 mm, 2.1 × 150 mm, Waters) and a gradient mobile phase consisting of water with 50 mM ammonium acetate (phase A) and acetonitrile (phase B) were used for chromatographic. Details of the chromatographic conditions have been previously described and can be found elsewhere [13].

### 2.4. Statistical Analysis

Categorical variables are described using frequencies and percentages while continuous variables are described using median and the percentiles 25 and 75. A non-parametric Mann–Whitney U test was used to assess the differences in gut-derived metabolites between the control and VTE groups, and a Wilcoxon signed rank test was used to assess differences in time (before–after) within groups. Correlations between continuous variables were conducted using Spearman’s Rho correlation; *p*-values < 0.05 were considered statistically significant. Data analysis was performed using SPSS 26.0 (IBM Corp, New York, NY, USA).

## 3. Results

### 3.1. Study Cohort Characteristics

A total of 111 individuals were enrolled. This included 54 individuals with VTE—22 males (40.7%) and 32 females (59.3%)—with a median age of 61.5 (47–78) years, and 57 control subjects—23 males (40.4%) and 34 females (59.6%)—with a median age of 64 (48–75) years. Regarding the follow-up time between the two groups, no differences were observed between control and VTE groups (*p* = 0.764). No differences were observed regarding gender distribution, age range, body mass index (BMI), and the presence of other comorbidities. There was a slightly significant increase in the number of patients treated with anti-platelet drugs in the VTE group. No differences were found in platelet count and hepatic function between the two groups. A significant decrease in PFA and estimated glomerular filtration rate (eGFR) was observed in VTE patients compared to the control group (Table 2).

### 3.2. Gut-Related Metabolites and VTE

**Figure 1:**
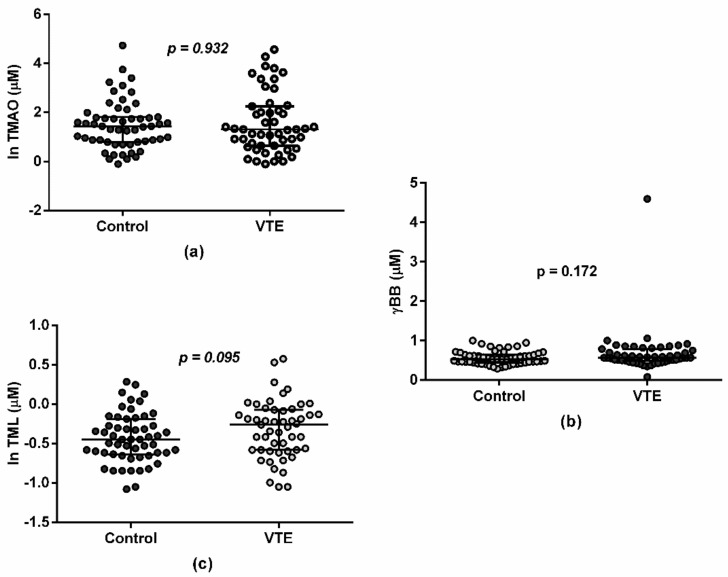
Plasma in TMAO (**a**), γBB (**b**), and TML (**c**) concentrations at baseline. Values are represented as median and interquartile range in both the control (n = 57) and VTE (n = 54) groups in a log-transformed way in order to facilitate visualization.

Regarding gut-derived metabolites and the presence/absence of VTE, no differences were found in TMAO, γBB, or TML concentrations between the control and VTE groups in the baseline measurement (*p* = 0.932, *p* = 0.172, and *p* = 0.095, respectively; Figure 1). Similarly, no differences were found for TMAO, γBB, and TML concentrations between the control and VTE groups in endpoint measurement (*p* = 0.346, *p* = 0.354, and *p* = 0.872, respectively; figure not shown).

### 3.3. Gut-Related Metabolites and Coagulation Parameters

For thrombosis-associated parameters, samples collected at baseline presented with a significant positive correlation between TMAO and RIS (R Spearman = 0.219, *p* = 0.024) and between TML and VWF (R Spearman = 0.194, *p* = 0.046). In these same samples, the most significant association with gut microbiota metabolites was found in parameters measuring thrombin generation, such as LAG, TTP, PEAK, and ETP. As shown in Table 3, TMAO, γBB, and TML showed a significant positive correlation with LAG and TTP. Conversely, TMAO, γBB, and TML presented a significant negative correlation with PEAK. Only TMAO was negatively associated with ETP. These correlations were maintained after analyzing controls and VTE patients separately, as shown in Supplementary Table S1.

For samples collected at endpoint, no correlations were found between platelet adhesiveness function tests measured as platelet AS, SC, or OB and gut-derived metabolites (data not shown). However, a slightly negative correlation between TMAO and AS was observed (R Spearman = −0.286; *p* = 0.004). In these same samples, when searching for correlation among gut-derived metabolites and PHR, no correlations were found except for a mild significant one between γBB and PHR at endpoint (*p* = 0.037).

The platelet function analysis, measured as PFA_EPI, was not significantly correlated with gut-related metabolites measured at baseline: TMAO (R Spearman = −0.113, *p* = 0.252), γBB (R Spearman = −0.116, *p* = 0.241), and TML (R Spearmen = −0.156, *p* = 0.113); and at the endpoint: TMAO (R Spearman = −0.023, *p* = 0.811), γBB (R Spearman = −0.009, *p* = 0.925), and TML (R Spearmen = −0.107, *p* = 0.274). PFA_ADP at both time points presented with similar non-significant results (data not shown). Other thrombosis-associated parameters (RIS, VWF, and disintegrin-like and metalloprotease with thrombospondin type 1 motif no. 13 -ADAMTS13-) did not present an association with TMAO, γBB, and TML (data not shown). 

### 3.4. Gut-Derived Metabolites over Time

The median (interquartile range) plasma concentrations for TMAO, γBB, and TML at baseline were 3.75 (2.08–7.53) μmol/L, 0.56 (0.46–0.70) μmol/L, and 0.67 (0.54–0.86) µmol/L, respectively, and 3.5 (2.1–6.8) μmol/L, 0.5 (0.49–0.70) μmol/L, and 0.66 (0.56–0.84) μmol/L, respectively, at endpoint. No significant differences were observed regarding TMAO, γBB, and TML concentrations between the two blood withdrawals (*p* = 0.462, *p* = 0.223, and *p* = 0.999, respectively) or when the control and VTE patients were analyzed separately (data not shown). Concerning TMAO, γBB, and TML correlations, a positive statistically significant correlation was observed for the three metabolites over time, as follows: TMAO at baseline vs. TMAO at endpoint (R Spearman = 0.326; *p* = 0.001), γBB at baseline vs. γBB at endpoint (R Spearman = 0.691, *p* = 0.001), and TML at baseline vs. TML at endpoint (R Spearman = 0.420; *p* = 0.001).

## 4. Discussion

The present work investigated the association between TMAO and two of its gut-derived intermediate metabolites (γBB and TML) with some hemostasis parameters used in clinical practice for the study of VTE risk.

Our initial hypothesis was based on previous studies indicating a relationship between TMAO and thrombosis in animal models [6,7,14,15] and in vitro studies [6,7]. It has been described previously that TMAO heightens thrombosis potential in mice by stimulating Ca^2+^ release from intracellular stores [6]. Specifically, the part played by TMAO and gut microbiota in increasing thrombosis potential in vivo has been supported by direct TMAO infusion and studies involving microbial transplantation [6]. The same authors showed that host hepatic FMO3 acts in diet-dependent and gut microbiota-dependent changes regarding platelet responsiveness and thrombosis potential in vivo [15]. Likewise, a previous work demonstrated that FMO3 knockout mice significantly reduced systemic TMAO concentrations and thrombosis potential [14]. An in vitro study relating TMAO and atherosclerotic thrombosis found that TMAO increased tissular factor activity and thrombin production [7]. Similarly, different works performed with inhibitors of microbial choline TMA lyase activity were able to suppress platelet aggregation [16,17]. In parallel, a work performed with germ-free mice colonized with microbiomes from low and high TMAO donors showed that TMAO is significantly associated with platelet aggregation responses [18].

In contrast to the above-mentioned works, no differences were found in our study regarding TMAO, γBB, and TML concentrations between the control and VTE groups at baseline and follow-up. This leads to the idea that, in the context of VTE, the implication of TMAO and its gut-derived metabolites may be limited, at least in our cohort of patients. This negative result agrees with the recent study of Reiner et al. in which no statistical association between TMAO and the risk of VTE recurrence in patients was found, despite demonstrating that TMAO presented with a significant U-shape association with all-cause mortality [10]. To our knowledge, no other work regarding the association of TMAO and related metabolites with VTE patients has been performed.

Regarding the hemostasis parameters, only several mild correlations were found in our cohort of patients between the microbiota metabolites and platelet function or thrombosis-associated parameters. These include a mild positive correlation for samples measured at baseline for RIS and VWF for TMAO and TML, respectively. Some correlations were also observed for parameters evaluating thrombin generation: LAG and TTP positively correlated with TMAO, TML, and γBB; PEAK negatively correlated with TMAO, TML, and γBB; and ETP negatively correlated with TMAO. These negative correlations are in the opposite direction of what would be expected if TMAO and its derivatives were related to VTE risk. Thus, the obtained results are not in line with the initial hypothesis of an association between TMAO and VTE, regardless of the numerous human studies relating TMAO to the impairment of coagulation parameters. A previous work with a cohort of 1627 patients found a weak correlation of TMAO with markers of platelet activation and showed that platelet reactivity and TMAO could be used as a mortality predictor even after adjusting for confounding factors [19]. Interestingly, the study found no significant interaction between platelet reactivity and TMAO for all-cause mortality and CVD mortality, suggesting their independence. Another study found that a 2-month oral choline supplementation given to humans caused an increased dose-dependent platelet aggregation response that could be attenuated by aspirin treatment [8]. Additionally, in patients with atrial fibrillation and thrombus formation, TMAO significantly correlated to platelet aggregation [20]. Conversely, another study of untreated HIV patients found no association between TMAO and platelet function [21].

Regarding TMAO and its derivative metabolite measurement over time, TMAO has been previously described as stable when stored at −80 °C for a period of five years, despite multiple freeze–thaw cycles [22]. The first study regarding intra-individual variation of TMAO over time was performed on type 2 diabetic patients; TMAO was evaluated every six months for two years. The study found a reliability coefficient of 0.17 with a coefficient variation of 63.3% [23]. Our results are in line with previous studies performed over seven years [24] and one year [25], demonstrating the high within-individual variability in plasma TMAO concentrations and supporting the usefulness of working with serial measures of these metabolites.

Several limitations should also be considered. Due to technical limitations, it was not possible to determine all coagulation parameters at both sampling times. Platelet function analysis, platelet hyperreactivity, and thrombin generation parameters were only analyzed in samples obtained at baseline, whereas platelet adhesiveness and thrombosis-associated parameters were only analyzed in samples obtained at endpoint. It can neither be ruled out that individual factors such as dietary habits or physical activity may have influenced the studied parameters over time, thus limiting our analysis with respect to the time when the VTE event occurred. This limitation is, however, difficult to overcome because the acute response has a major influence over many aspects of metabolism and might therefore have a major influence on TMAO concentration during the acute phase of VTE. In fact, a small study by Matsuzawa et al. [26] demonstrated that plasma TMAO concentrations are lower in the acute phase compared to the chronic phase of ST elevated myocardial infarction and are thus unrelated to recurrence risk.

Similar to the results by Reiner et al. [10], our work shows that TMAO concentrations and those of gut related metabolites are not good predictors of risk of VTE. Despite including a reduced number of patients, our work presents with several advantages over the study by Reiner et al.: first of all, it compares VTE patients vs. controls; second, it includes younger patients; and third, it evaluates a wide set of coagulation parameters. However, and due to the limited number of included patients, our results should be interpreted cautiously. A replication study in a larger population would strengthen the observed results. Additionally, further studies evaluating the effect of the acute phase on TMAO concentrations would be appropriate. Nonetheless and in view of the current evidence, the analysis of TMAO does not seem to be of interest to establish the risk of VTE in clinical practice.

## 5. Conclusions

No differences were found regarding TMAO, γBB, and TML concentrations between the control and VTE groups. Mild correlations were found in our VTE cohort between the microbiota metabolites and some of the coagulation parameters studied. In all cases, the correlations were mild or went in the opposite direction of what would be expected if TMAO and its derivatives were related to VTE risk.

## Figures and Tables

**Table 1:** Characteristics of the consecutive thrombotic events.

	Spontaneous			Non-Spontaneous		
	Female	Male	Total	Female	Male	Total
Isolated deep vein thrombosis (n, %)	11 (55.0)	8 (50.0)	19 (52.8)	5 (41.7)	2 (33.3)	7 (38.9)
No isolated deep vein thrombosis (n, %)	2 (10.0)	1 (6.3)	3 (8.3)	4 (33.3)	3 (50.0)	7 (38.9)
Isolated pulmonary Embolism (n, %)	6 (30.0)	6 (37.5)	12 (33.3)	3 (25.0)	1 (16.7)	4 (22.2)
Visceral thrombosis (n, %)	-	1 (6.3)	1 (2.8)	-	-	-
Venous sinus thrombosis (n, %)	1 (5.0)	-	1 (2.8)	-	-	-
Total	20 (100)	16 (100)	36 (100)	12 (100)	6 (100)	18 (100)

**Table 2:** Clinical and biochemical parameters for the control and VTE groups.

	Control (n = 57)	VTE (n = 54)	*p* Value
Age at baseline (y)	64 (48–75)	61.5 (46.8–78)	0.873
Gender (% males)	40.4	40.7	0.967
BMI (Kg/m^2^)	26 (24.2–29)	27.6 (24.2–29.7)	0.335
Smoking (n, %)	10 (17.5)	10 (18.5)	0.894
Alcohol consumption (n, %)	28 (49.1)	26 (48.1)	0.918
Hypertension (n, %)	23 (40.4)	26 (48.1)	0.408
Dyslipidemia (n, %)	19 (33.3)	17 (31.5)	0.835
Statins (n, %)	14 (24.6)	14 (25.9)	0.869
Diabetes mellitus (n, %)	7 (12.3)	2 (3.7)	0.098
Autoimmune disease (n, %)	7 (12.3)	5 (9.3)	0.608
Arterial thrombosis background (n, %)	-	2 (3.7)	0.239
Anti-platelet drugs (n, %)	2 (3.5)	8 (14.8)	0.049
PFA_ADP (s)	82 (72–96.8)	59 (54.8–63)	<0.0001
PFA_EPI (s)	116.5 (97.8–137.8)	83 (77–88)	<0.0001
Platelet count (×10^9^/L)	234 (204–270.5)	234 (203–268.8)	0.750
eGFR (mL/min/1.73 m^2^)	85.4 (66.7–90)	70.8 (60–90)	0.012
ALT (IU/L)	18 (15–24)	20 (16–35.5)	0.060
AST (IU/L)	19 (16–21)	19 (16–21)	0.266

PFA_ADP: platelet function analysis adenosine 5′-diphosphate; PFA_EPI: platelet function analysis epinephrine; BMI: body mass index; eGFR: estimated glomerular filtration rate; ALT: alanine amino transferase; AST: aspartate amino transferase. Results are expressed as median (P_25_–P_75_). A Mann–Whitney U test was performed to compare differences between the control and VTE groups.

**Table 3:** Spearman correlation test for TMAO, γBB, and TML and thrombin generation parameters (LAG, TTP, ETP, and PEAK).

	LAG (min)	TTP (min)	ETP	PEAK
TMAO	0.220 (*p* = 0.024)	0.285 (*p* = 0.003)	−0.209 (*p* = 0.033)	−0.33 (*p* = 0.001)
γBB	0.207 (*p* = 0.035)	0.259 (*p* = 0.008)	−0.042 (*p* = 0.673)	−0.212 (*p* = 0.031)
TML	0.228 (*p* = 0.02)	0.205 (*p* = 0.036)	−0.124 (*p* = 0.206)	−0.195 (*p* = 0.046)

LAG: lag time; TTP: time to peak; ETP: endogenous thrombin potential; PEAK: peak height. Data are presented as R Spearman correlation and *p* value.

## Data Availability

The data that support the findings of this study will be available to other researchers upon reasonable request.

## Supplementary Materials

The following supporting information can be downloaded at: https://www.mdpi.com/article/10.3390/jcm11051425/s1, Table S1: Spearman correlation test for TMAO, γBB, and TML and thrombin generation parameters (LAG, TTP, ETP, and PEAK) for controls and VTE patients separately.
