# Supplementary material for: TMAO and Gut Microbial-Derived Metabolites TML and γBB Are Not Associated with Thrombotic Risk in Patients with Venous Thromboembolism
Source: J Clin Med. 2022 Mar 4;11(5):1425. doi: 10.3390/jcm11051425 (PMC8911412; doi:10.3390/jcm11051425)
Supplement: Supplementary file 1 [file jcm-11-01425-s001.zip › jcm-1595828-supplementary.pdf]

Supplementary Table S1. Spearman correlation test for TMAO, γBB, and TML and thrombin generation parameters (LAG, TTP, ETP, and PEAK) for controls and VTE patients separately

		LAG (min)	TTP (min)	ETP	PEAK
TMAO	Control	0.039 (p = 0.775)	0.155 (p = 0.258)	-0.082 (p = 0.553)	-0.217 (p = 0.112)
	VTE	0.383 (p = 0.006)	0.390 (p = 0.005)	-0.353 (p = 0.012)	-0.426 (p = 0.002)
γBB	Control	0.170 (p = 0.215)	0.168 (p = 0.220)	-0.074 (p = 0.589)	-0.139 (p = 0.311)
	VTE	0.236 (p = 0.102)	0.363 (p = 0.010)	0.017 (p = 0.909)	-0.299 (p = 0.037)
TML	Control	-0.146 (p = 0.287)	-0.046 (p = 0.739)	-0.074 (p = 0.589)	-0.062 (p = 0.654)
	VTE	0.550 (p < 0.0001)	0.486 (p < 0.0001)	-0.266 (p = 0.062)	-0.406 (p = 0.003)

LAG: lag time; TTP: time to peak; ETP: endogenous thrombin potential; PEAK: peak height.
Data are presented as R Spearman correlation and p value
